# Effect of feeding of sorghum stover based complete feed blocks containing guar meal as protein supplement on reproductive efficiency, hormone profile and antioxidant status in Deccani ewes

**DOI:** 10.1371/journal.pone.0268893

**Published:** 2022-05-26

**Authors:** B. Vidya, M. Venkateshwarlu, D. Nagalakshmi, A. Sarat Chandra, V. Chinni Preetham, N. Nalini Kumari

**Affiliations:** 1 Department of Livestock Farm Complex, College of Veterinary Science, Rajendranagar, PVNR TVU, Hyderabad, India; 2 Department of Animal Nutrition, College of Veterinary Science, Rajendranagar, PVNR TVU, Hyderabad, India; 3 Department of Livestock Production Management, College of Veterinary Science, Rajendranagar, PVNR TVU, Hyderabad, India; 4 Department of Poultry Science, College of Veterinary Science, Rajendranagar, PVNR TVU, Hyderabad, India; 5 Department of Animal Nutrition, College of Veterinary Science, Korutla, PVNR TVU, Jagtial, India; University of Life Sciences in Lublin, POLAND

## Abstract

Guar meal (GM) can be considered as an alternative protein source for the livestock and has a potential value as a feed to animals with a high protein content ranging between 45–55%. A study was conducted to evaluate the effect of partial replacement of groundnut cake (GNC) with guar meal (GM) on reproductive efficiency, hormone profile, antioxidant status in Deccani ewes fed sorghum stover based complete feed blocks (SSCFB). Twenty-one non-pregnant Deccani ewes (b. wt. 23.34 ±0.40 kg; 2–4 years old and body condition score 2.51±0.56) were randomly divided into three groups to contain 7 animals each in a completely randomized design. Three dietary treatments viz. S1: conventional feeding (sorghum stover and concentrate mixture fed separately in 50:50 ratios to meet the requirement), S2: SSCFB with GNC as protein supplement in concentrate mixture and S3: SSCFB with GM replacing GNC nitrogen of S2 at 50% level. The ewes were synchronised for estrus with progesterone impregnated intravaginal sponges and naturally mated. The ewes were fed at the rate of 3.1 and 3.7% of their body weight from 1^st^ day of experiment—to 108^th^ day of gestation and from 108^th^ day of gestation to till lambing, respectively. The replacement of 50% of GNC nitrogen with GM and densification of diet had no effect (P>0.05) on average DMI (g), antioxidant status, progesterone concentration, conception rate, non return rate, no of matings per service, lambing rate, body weights of lambs at birth, 15 and 30 days of age. GNC can be partially replaced by GM in the ewes diet without any adverse effect on reproductive parameters, antioxidant status, progesterone concentration and weight of lambs.

## Introduction

Effective utilization of available feed resources is the key for economical livestock rearing. The utilization of crop residues for increased animal production has received greater research attention within the past few decades because of the higher quantities of crop residues production. The crop residues predominantly roughages, however are of inferior quality, but owing to several constraints, developing countries like India even today have to depend largely upon these crop residues for feeding ruminant population. A possible and perhaps the most viable proposition could be the complete feed block (CFB) technology which would enable the livestock farmer to utilize these resources more effectively resulting in better performance of the animals. The complete feed blocks provide optimum protein and energy apart from supplying adequate amount of minerals and vitamins for the animals, thus enhances reproductive efficiency. The CFB technology also provides an opportunity for the incorporation of unconventional feed ingredients such as guar meal in diets.

Protein is the second most expensive nutrient after energy. Groundnut cake, cotton seed cake and soybean meal have conventionally been the main protein sources in ruminant feeds but there exists a major gap between the demand and supply of these conventional protein resources for livestock feeding in the world. In order to fill this gap of demand and supply, it is essential to exploit the use of non-conventional protein feed resources in ruminant production systems. Guar meal (*Cyamopsis tetragonoloba*) can serve as a promising alternative source of protein for the animal feed industry. Guar meal (GM) after gum extraction is a potential source of protein and contains about 40–52% crude protein [1]. However, the use of guar meal in livestock diets is limited because it contains potent antinutritional factors such as residual gum, saponins trypsin inhibitors, haemagglutinin and extractable polyphenols. Excessive levels of these antinutritional factors can cause bitterness, reduce nutrient availability, palatability and induce toxicity in animals when consumed beyond threshold levels [2]. Prolonged feeding of unconventional feeds containing high levels of anti-nutritional compounds adversely affects the nervous, immune, endocrine and reproduction systems of the animals. Various studies [3–7] have investigated the effect of feeding of guar meal on growth performance, digestibility of nutrients and carcass characteristics in ruminants but none has investigated the effects of guar meal on the reproductive characteristics of livestock, or whether its use in the long term can cause any adverse effects on ewe reproductive performance. Therefore, the aim of the present study was to evaluate the effect of partial replacement of groundnut cake with guar meal and also to study the effect of densification of complete feed on reproductive performance, hormone profile and antioxidant status in Deccani sheep.

## Materials and methods

### Animals, experimental design and feeding management

Animal handling techniques and experimental protocols followed in the present study were as per the guidelines issued by Committee for the Purpose of Control and Supervision of Experiments on Animals (CPCSEA). Twenty one non-pregnant (2–4 years of age), healthy, cycling, multiparous ewes, which were homogenous in weight (23.52 ±0.41 kg) and body condition score (2.51±0.56), were selected for the study. The selected ewes were randomly divided into three groups to contain 7 animals each in a completely randomized design. Three dietary treatments were S1: Conventional feeding where sorghum stover and concentrate mixture was offered separately in 50:50 ratio to meet the ICAR (2013) [8] nutrient requirement of the ewes, S2: Sorghum stover based complete feed blocks (SSCFB) with groundnut cake as protein supplement in concentrate mixture and S3: SSCFB with guar meal replacing groundnut cake nitrogen of S2 at 50% level. First, Sorghum stover based complete diets (50R: 50C) was formulated to meet the nutrient requirements of ewes as per the recommendations of ICAR (2013) [8] and then densified complete feed blocks were prepared by compacting the mash materials in an iron mould into sizes of 15 x 15 x 10 cm using a specially designed semi-automatic hydraulic press fitted with a manual ejection system at a pressure of 1500 psi. In case of conventional feeding (S1), the ewes were fed sorghum stover and concentrate mixture (18.01% crude protein and 71.66% Total digestible nutrients) separately in 50:50 ratios to meet the nutrient requirements. The ingredient composition of experimental diets is presented in Table 1.

**Table 1:** Ingredient composition (%) of experimental diets for reproductive study.

Name of the ingredient (%)	S2	S3	Concentrate mixture
Sorghum stover	50.0	50.0	0.0
Maize grain	7.4	8.4	44.2
Groundnut cake	9.0	4.5	25.2
Deoiled rice bran	25.0	25.0	30.0
Guar meal	0.0	3.5	0.0
Molasses	8.0	8.0	0.0
Mineral mixture	0.2	0.2	0.2
Salt	0.4	0.4	0.4
Total	100.0	100.0	100.0

S2: Sorghum stover based complete feed blocks (SSCFB) with groundnut cake as a protein supplement.

S3: SSCFB with guar meal replacing groundnut cake nitrogen at 50% level.

All the ewes were housed in a well-ventilated animal shed with provision for individual feeding and watering. Deworming was done with niclosamide for parasites before the start of the experiment and subsequently at bi-monthly interval. All the ewes were vaccinated against sheep pox, PPR and enterotoxaemia as per the schedule and gradually adapted to experimental diets for about 10 days prior to start of the experiment. During first phase i.e., from 1^st^ day of experiment to the 108^th^ day of gestation, all the ewes were fed the respective diets at 3.1 per cent of their body weights. In second phase i.e., from 108^th^ day of gestation (second trimester) to till lambing all the animals were fed respective diets at 3.73 per cent of their body weight to meet the nutrient requirements as per ICAR (2013) [8]. Animals were fed experimental diets twice a day i.e., at 10.00 AM and 2.30 PM in equal proportions.

Daily feed intake of each ewe was recorded as the difference between weight of the feed offered and residues left. The ewes were weighed fortnightly using an electronic digital balance before offering feed and water in the morning. The feed intake was adjusted at fortnight interval according to body weight changes. Fresh drinking water was made available at all the times.

### Estrus synchronization and pregnancy diagnosis

Estrus synchronization began on the 15^th^ day after the start of the experiment. Estrus was induced in all the females using an intravaginal sponge containing 350 mg of natural progesterone (CSWRI sponges, Avikanagar, Rajasthan, India). The sponge was allowed to remain in the vagina for the next 12 days. Forty eight hours prior to sponge removal, each animal received an intramuscular injection of 500 IU of pregnant mare serum gonadotrophin (PMSG). Twenty four hours after the sponge removal an apronized ram was left along with the treated animals 3 times a day with each session lasting for 10 to 20 minutes to identify estrus. An ewe was considered to be positive for estrus when mounted by the ram and was in standing position. Later the ewe in estrus was separated and allowed for mating with a fertile ram. Ram to ewe ratio for mating was 1:3. Pregnancy diagnosis was conducted after 30 days of mating with a B-mode transrectal ultra-sonographic scanner with a 7.5 MHz transducer (ALOKA, Prosound 2).

The following reproductive parameters were measured: Onset of estrus (time taken for the ewes to exhibit estrus after the removal of sponge), estrous response, non-return rate, conception rate, no of matings per conception, gestation length, lambing rate, litter size and weights of lambs at birth, 15^th^ and 30^th^ day of age. In addition, daily feed intake and fort night body weights of the ewes were also evaluated. Antioxidant activity and progesterone concentration in serum was determined at different periods.

### Sample collection

Blood was collected from individual ewes on 15^th^ and 90^th^ day of pregnancy and 15 days before expected day of lambing (15d BL). Blood samples were collected aseptically from jugular vein of ewes into 5 ml vacutainers (Becton Dickinson) and kept in slanted position at room temperature for separation of serum. The serum samples were centrifuged at 3000 rpm for 5 minutes and transferred to 5 ml Eppendorf tubes and stored at -20°C for estimation of glutathione peroxidase activity, lipid peroxidation and serum progesterone concentration. Only serum without lipaemia was analysed. Blood analysis was completed within 2 days after collection.

The amount of feed offered and residue left after 24h from all the animals were collected and weighed separately and these were analyzed every fortnight for calculation of DMI.

### Analytical methods

The glutathione peroxidase activity was determined by the method proposed by Paglia and Valentine (1967) [9] with little modifications. Lipid peroxidation activity in serum was determined by the method of Ohkawa *et al*. (1979) [10]. The progesterone in the blood serum was estimated according to procedure given by Radwanska *et al*. (1978) [11] using ELISA kit supplied by Cal biotech Inc., El Cajon, CA, USA. ELISA Kit was validated by testing the progesterone concentration in the blood collected from healthy ewes.

The feed samples were subjected to proximate analysis and fibre fractions as per the methods described by AOAC. (1997) [12] and Van Soest *et al*. (1991) [13].

### Site of study and ethical statement

The experiment was carried out at the College of Veterinary Science, PVNR Telangana Veterinary University, Rajendranagar, Hyderabad (17°12’ N, 78°18’ E, 545 m above sea level) in India. The ambient temperature and relative humidity values during the period of study were in the range of 31–38°C and 28–39%, respectively. The experimental protocols were done with the approval of Institute’s Animal Ethics Committee (IAEC) of PVNR Telangana Veterinary University, Hyderabad, 500 030, Telangana, India.

### Statistical analysis

The results obtained in four phases of experiments were subjected to analysis through software (Version 15.0; SPSS) by applying one-way and two way multivariant analysis of variance through generalized linear model (antioxidant enzyme activity and serum progesterone concentration). The treatment means were ranked using Duncan’s multiple range test with significance at 5% level [14]. All the statistical procedures were done as per Snedecor and Cochran (1994) [15]. The results of reproductive parameters were analyzed by using online Chi-square calculator (www.socscistatistics.com) with significance at 5% level, in a 2×3 contingency table.

## Results

### Feed and dry matter intake

**Fig 1:**
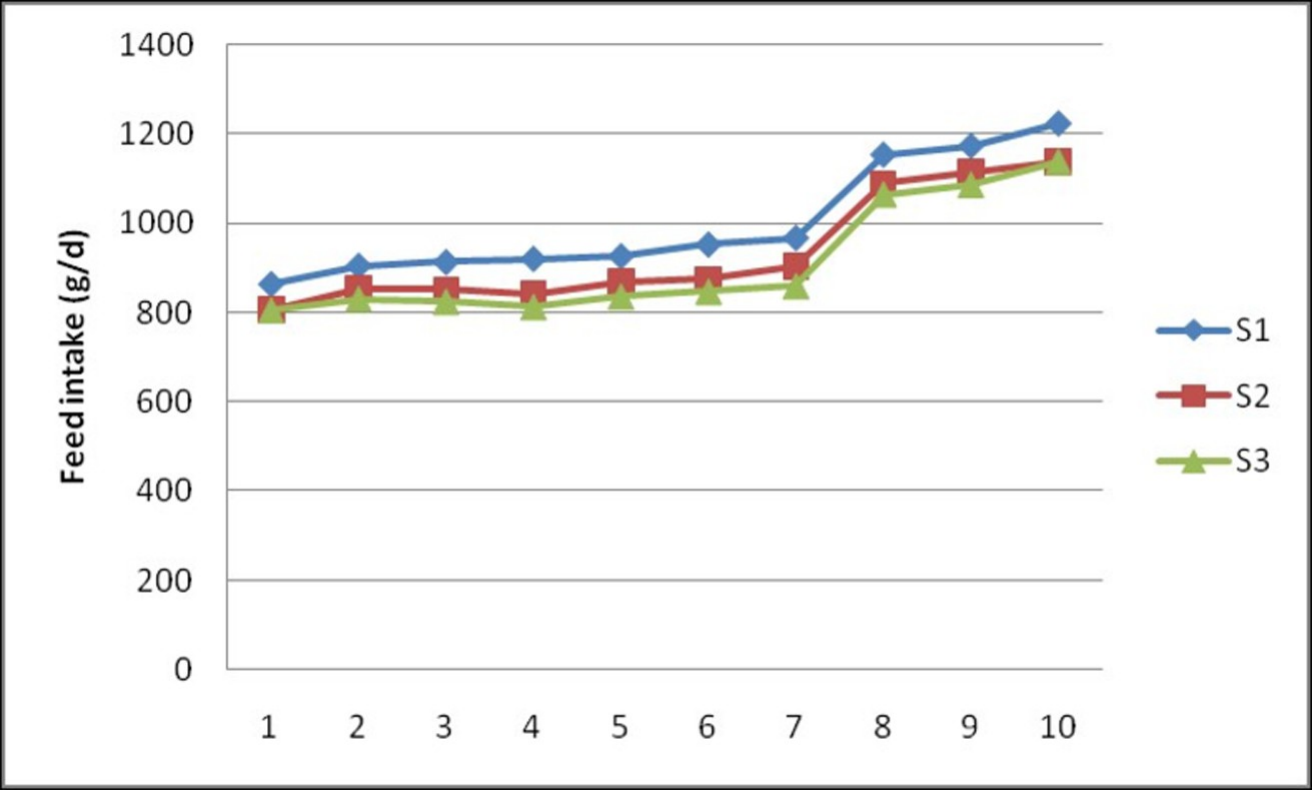
Effect of feeding of sorghum stover based complete feed blocks containing guar meal on fortnightly daily feed intake (g/day) in Deccani ewes.

**Fig 2:**
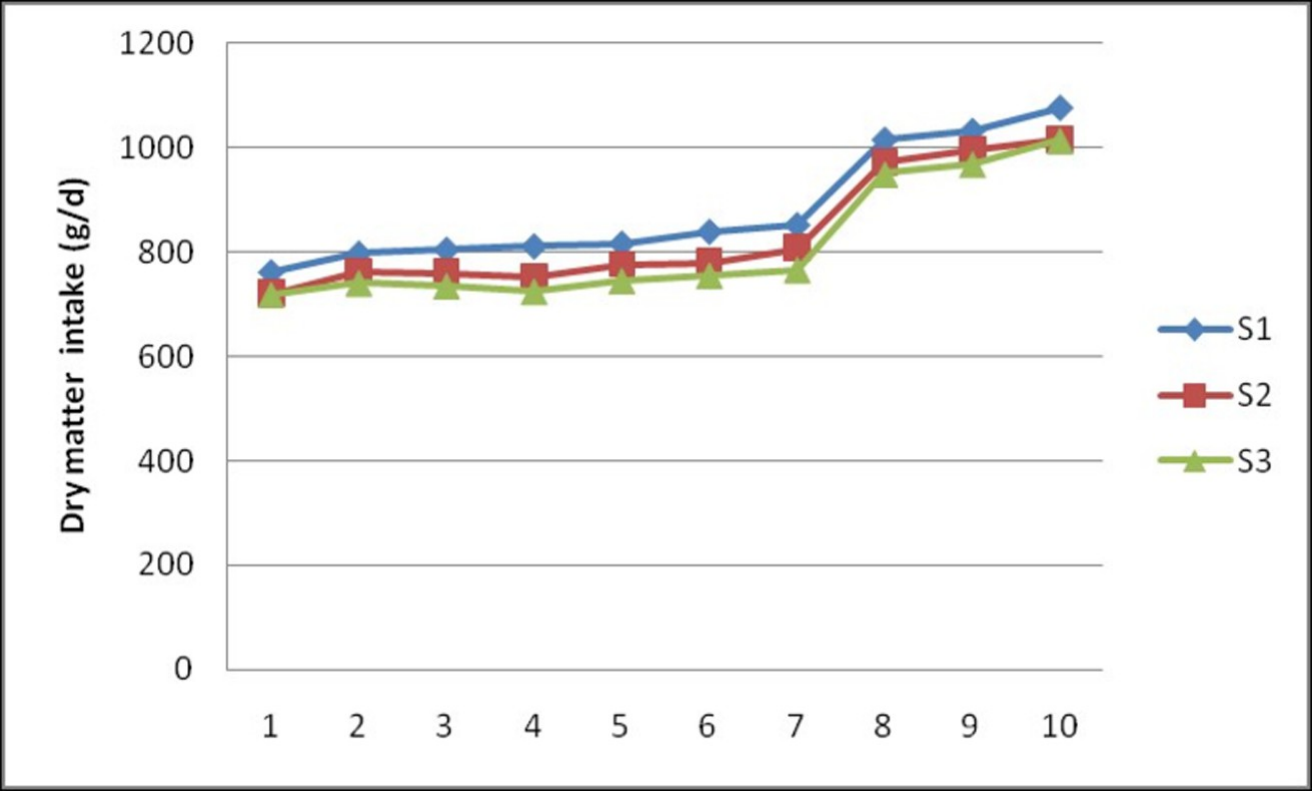
Effect of feeding of sorghum stover based complete feed blocks containing guar meal on fortnightly daily dry matter intake (g/day) in Deccani ewes.

The chemical composition of experimental diets used in the study is presented in Table 2. The feed intake (g/day) (Fig 1) and DMI (g/d) (Fig 2) in S1, S2 and S3 diets ranged from 864.7± 35.22 to 920.9 ± 39.36 and 760.9± 30.99 to 810.4± 34.64; 806.7± 24.78 to 843.6 ± 21.97 and 720.3± 22.13 to 753.3±19.61 and 804.0±11.41 to 811.4±24.28 and 717.9±10.19 to 724.5±21.68, respectively from 1^st^ to 4^th^ fortnight. By the time of 10^th^ fortnight ewes fed S1, S2 and S3 diets consumed 1222.38±39.18, 1137.71±55.17 and 1136.11±45.41 g feed and 1075.70±34.41, 1015.81±49.26 and 1014.40±40.55 g of dry matter on average, respectively. During trial period, feeding of ewes with sorghum stover based diets in block form had no effect (P>0.05) on feed and DM intake. Similarly, dietary inclusion of guar meal did not show any effect (P>0.05) on feed and DM intake in ewes.

**Table 2:** Nutrient composition of experimental diets used in reproduction study (% on DMB).

Constituent	Complete diets (SSCFB)		Sorghum stover	Concentrate mixture
	S2	S3		
**Proximate constituents**				
Dry matter	91.00	90.00	90.50	89.80
Organic matter	90.51	90.54	90.29	92.82
Crude protein	10.22	10.31	4.39	18.01
Ether extract	2.69	2.48	0.93	4.03
Crude fibre	24.82	24.73	37.13	9.43
Nitrogen free extract	52.77	53.01	47.84	61.33
Total ash	9.49	9.46	9.71	7.18
**Cell wall constituents**				
Neutral detergent fibre	51.97	52.29	74.80	27.82
Acid detergent fibre	32.39	32.28	55.13	15.41
Hemicellulose	19.58	20.01	19.67	12.40
Cellulose	22.98	21.25	45.60	6.10
Acid detergent lignin	5.59	5.49	6.17	3.89

Each value is the average of duplicate analysis.

On dry matter basis except for dry matter.

S2: Sorghum stover based complete feed blocks (SSCFB) with groundnut cake as a protein supplement.

S3: SSCFB with guar meal replacing groundnut cake nitrogen at 50% level.

### Body weight changes in ewes

**Fig 3:**
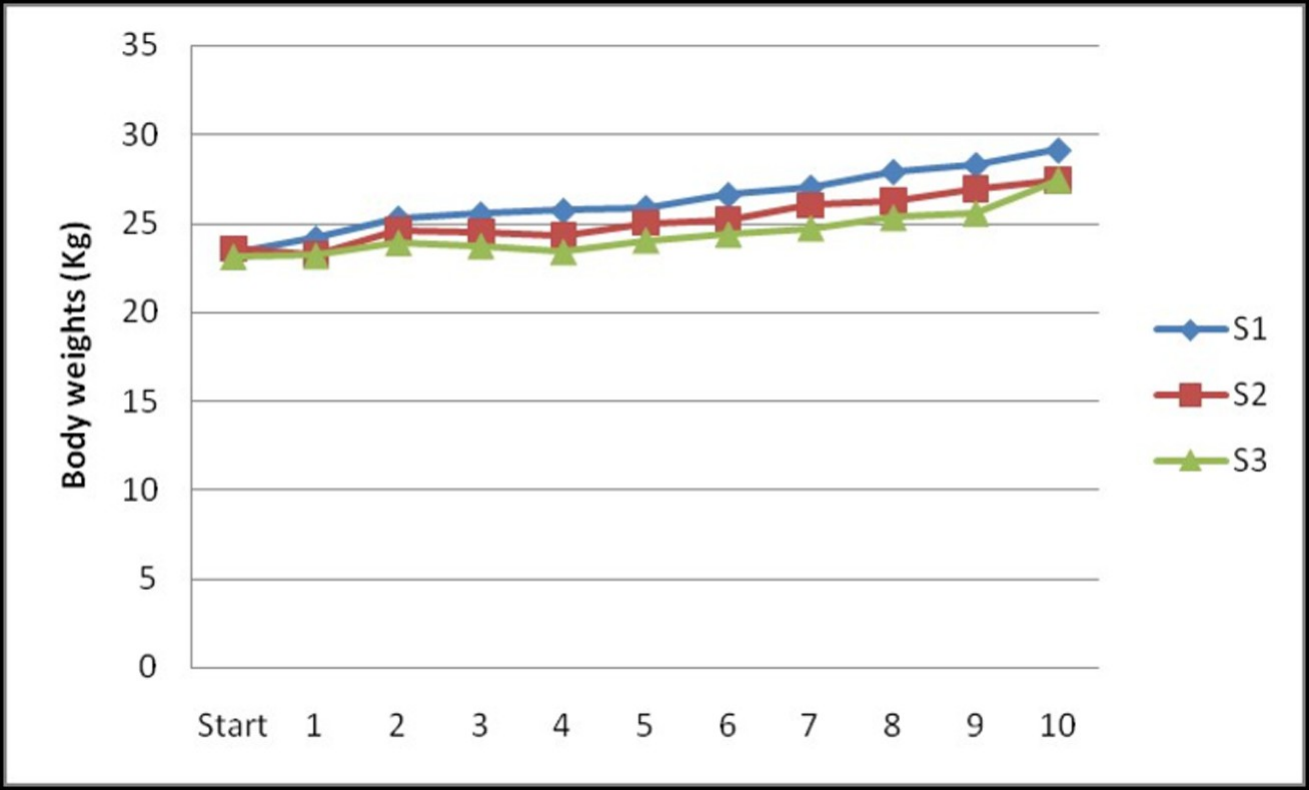
Effect of sorghum stover based complete feed blocks containing guar meal on fortnightly average body weights (kg) of Deccani ewes.

The initial body weights of the ewes fed S1, S2 and S3 diets were 23.34±0.99, 23.60±0.74 and 23.10±0.26 kg, respectively (Fig 3) and the corresponding weights at the end of 10^th^ fortnight was 29.12±1.01, 27.45±1.33 and 27.42±1.10 kg, respectively. Statistically, there was no significant difference in fort night body weight changes (kg) in ewes fed S1, S2 and S3 diets from first to 10^th^ fortnight.

### Antioxidant enzyme activity

The lipid peroxidation (nanomol/ml) and glutathione peroxidase (U/dl) concentrations assessed on 15^th^ and 90^th^ day of pregnancy and at 15 d before expected day of lambing in ewes fed S1, S2 and S3 diets are presented in Table 3. The lipid peroxidation, irrespective of diets was significantly (P<0.01) varied and lipid peroxidation was higher (P<0.01) on 90^th^ day of pregnancy, lowest (P<0.01) on 15^th^ day of pregnancy and intermediary on 15 d before expected day of lambing. However, there was no significant difference among the ewes fed different experimental diets. No interaction was observed between the diet and period in lipid peroxidase concentration.

**Table 3:** Effect of feeding of sorghum stover based complete feed blocks containing guar meal on antioxidant status and serum progesterone in Deccani ewes.

Diet	Period				P-value		
	15d	90d	15d BL	Mean ±SE	D	P	DxP
**Lipid peroxidation (nanomol/ml)**							
**S1**	2.32±0.02	3.73±0.05	3.25±0.01	3.10±0.14	0.244	0.001	0.765
**S2**	2.36±0.01	3.77±0.03	3.31±0.04	3.14±0.14			
**S3**	2.37±0.01	3.70±0.02	3.27±0.03	3.11 ±0.13			
**Mean ± SE**	2.35 ^C^±0.01	3.73^A^±0.020	3.28^B^±0.02				
**Glutathione peroxidase (U/dl)**							
**S1**	418.33±4.90	559.33±2.30	360.67±1.15	446.11^b^±19.74	0.002	0.001	0.809
**S2**	425.83±3.21	568.50±3.73	369.17±2.51	454.50^a^±19.84			
**S3**	417.17±4.15	562.67±2.92	356.83±2.32	445.56^b^±20.43			
**Mean ±SE**	420.44^B^±2.43	563.50^A^±1.88	362.22^C^±1.68				
**Serum progesterone (ng/ml)**							
**S1**	7.41±0.20	24.27±0.82	32.59±0.53	21.42±2.49	0.199	0.001	0.066
**S2**	7.13±0.18	23.29±0.38	30.38±1.45	20.27±2.34			
**S3**	7.07±0.10	21.96±0.77	33.07±0.50	20.70±2.53			
**Mean ± SE**	7.20^C^**±**0.10	23.17^B^**±**0.44	32.01^A^**±**0.58				

S1: Conventional feeding (sorghum stover and concentrate mixture fed separately in 50:50 ratios to meet the requirement).

S2: Sorghum stover based complete feed blocks (SSCFB) with groundnut cake as a protein supplement.

S3: SSCFB with guar meal replacing groundnut cake nitrogen at 50% level.

15d and 90d: Blood collected on 15^th^ and 90^th^ day of pregnancy.

15d BL: 15^th^ day from expected day of lambing.

SEM: Standard Error Mean; P value: Probability value.

^abc^Means with different superscripts in a column differ significantly: P<0.00^ABC^Means with different superscripts in a row differ significantly: P<0.001.

Glutathione peroxidase concentration was significantly (P<0.001) highest on 90^th^ day of pregnancy, lowest on 15 d before expected day of lambing and intermediary on 15^th^ day of pregnancy (Table 3). Irrespective of days of blood collection, highest (P<0.001) Glutathione peroxidase concentration was recorded in ewes fed S2 diet than other groups. The interaction between the diet vs period was not significant.

### Serum progesterone

The progesterone (ng/ml) levels irrespective of diets was higher (P<0.001) at 15 d before expected date of lambing, while the concentration was lower on day 15 and intermediate on 90^th^ day of pregnancy. Irrespective of days of blood collection, the overall progesterone concentration (P> 0.05) was comparable among the three diets (Table 3).

### Reproductive traits

Table 4 describes the effect of partial replacement of groundnut cake Nitrogen with guar meal on reproductive performance in Deccani ewes. The estrus response was defined as the percentage of animals identified in estrus by apronized ram following intravaginal sponge removal. All ewes maintained on S1, S2 and S3 diets were identified to be in estrus by the apronized ram after intravaginal sponge removal. Therefore, the estrus response was found to be 100% in all the groups (P>0.05).

**Table 4:** Effect of feeding of sorghum stover based complete feed blocks containing guar meal on reproductive traits of Deccani ewes.

Attribute	S1	S2	S3	SEM	P value
Estrus response (%)	100	100	100	0.000	1.000
Time taken for exhibition of estrus (h)	26.32±0.66	26.43±0.49	28.23±0.92	0.437	0.131
Non return rate (%)	85.71	100	85.71	6.717	0.932
Conception rate (%)	85.71	100	85.71	6.717	0.932
No. of matings per conception	1.0	1.0	1.0	0.000	1.001
Lambing rate per no. of ewes tupped (%)	85.71	100	85.71	6.717	0.932
Lambs born per total no. of ewes (%)	85.71	100	85.71	6.717	0.932
Litter size	1.0	1.0	1.0	0.000	1.001
Gestation length (days)	148.33±1.87	150.33±1.36	153.33±2.89	1.223	0.256

S1: Conventional feeding (sorghum stover and concentrate mixture fed separately in 50:50 ratios to meet the requirement).

S2: Sorghum stover based complete feed blocks (SSCFB) with groundnut cake as a protein supplement.

S3: SSCFB with guar meal replacing Groundnut cake nitrogen at 50% level.

SEM: Standard Error Mean; P: Probability value.

The onset of estrus after vaginal sponge removal was recorded as 26.32, 26.43 and 28.23 hours in ewes fed S1, S2 and S3 diets, respectively and the complete block feeding and replacement of 50% of groundnut cake -nitrogen with guar meal did not influence the time taken for the animals to exhibit estrus after the sponge removal.

The per cent non-return rate and conception rate were 85.71, 100 and 85.71 in ewes fed S1, S2 and S3 diets. There was no significant (P>0.05) difference among three groups.

The number of matings per conception did not differ significantly in ewes fed S1, S2 and S3 diets and was 1.00 for all experimental groups. The lambing rate was expressed as number of lambs born per number of ewes mated and was 100% in ewes fed S2 diet and in S1 and S3 diets was 85.71%. The per cent of lambs born to total number of ewes in group was 85.71, 100, and 85.71 in ewes fed S1, S2 and S3 diets, respectively. The lambing rate and per cent of lambs born did not differ significantly (P>0.05) in ewes fed S1, S2 and S3 diets.

The average litter size for all the three dietary groups was 1.0 and did not differ between three groups. The duration of gestation did not seem to be influenced by the dietary variation and was recorded as 148.33±1.87, 150.33±1.34 and 153.33±2.89 days in ewes fed S1, S2 and S3 diets, respectively.

### Body weight of lambs

The body weights (kg) of lambs were 2.23±0.13, 2.21±0.13 and 2.24±0.14 at birth and 3.56±0.23, 3.14±0.19 and 3.25± 0.24 on 15^th^ day and 5.08±0.22, 4.41±0.28 and 4.39±0.23 on 30^th^ day in S1, S2 and S3 groups, respectively (Table 5). The feeding of SSCFB with inclusion of GM had no effect (P>0.05) on lamb body weights at birth, on day 15^th^ and 30^th^ day.

**Table 5:** Effect of feeding of sorghum stover based complete feed blocks containing guar meal on lamb weights (kg) at birth, 15 and 30d of age.

Diet	Sex		Lamb wt.(kg)								
			At birth			At 15 days age			At 30 days age		
	F	M	F	M	Average	F	M	Average	F	M	Average
**S1**	2	4	2.25±0.20	2.21±0.20	2.23±0.13	3.62±0.22	3.49±0.34	3.56±0.23	5.06±0.03	5.09±0.34	5.08±0.22
**S2**	4	3	2.24±0.10	2.18±0.30	2.21±0.13	3.00±0.14	3.28±0.45	3.14 ±0.19	4.10±0.25	4.62±0.61	4.39 ±0.28
**S3**	3	3	2.04±0.13	2.43±0.22	2.24±0.14	3.15±0.51	3.35±0.11	3.25±0.24	4.21±0.47	4.52±0.14	4.39±0.23
N			9	10	19	9	10	19	9	10	19
SEM			0.073	0.130	0.051	0.189	0.176	0.125	0.225	0.229	0.159
P value			0.334	0.517	0.900	0.257	0.692	0.256	0.07	0.110	0.088

S1: Conventional feeding (sorghum stover and concentrate mixture fed separately in 50:50 ratios to meet the requirement).

S2: Sorghum stover based complete feed blocks (SSCFB) with Groundnut cake as a protein supplement.

S3: SSCFB with guar meal replacing Groundnut cake nitrogen at 50% level.

F: Female; M: Male.

N: Number of observations.

SEM: Standard Error Mean; P value: Probability value.

## Discussion

### Feed and dry matter intake

The results of the present study showed that inclusion of GM by replacing of 50% GNC nitrogen and feeding of complete diet in block form had no effect (P>0.05) on average DMI (Fig 2). The lack of effect on DMI might be due to the iso-nitrogenous diets [16] and it could also be attributed to the usage of toasted guar meal as processing removes beany odour, trypsin inhibitors and residual gum making more palatable and digestible. Our results are consistent with Janampet *et al*. (2016) [7] who reported that, the DMI was not affected with replacement of 50% GNC with GM. Similarly, no effect on DMI was also reported by Jongwe *et al*. (2014) [5] with 75% replacement of GNC with GM in Sahiwal cows and by replacement of soyabean meal with rapeseed meal in dairy cows [17]. Similar to the present study, no effect of densification on DMI was reported by Sharma *et al*. (2010) [18] in crossbred calves and Raju (2018) [19] in sheep.

### Body weight changes in ewes

No loss in BW of ewes was observed during the trial period and there was a gradual increase in BW in all groups throughout the experimental period (Fig 3). The gradual increase in BW observed from 6^th^ fortnight was due to usual gain in weight in pregnant animals because of foetal growth. The inclusion of guar meal had no adverse effect (P>0.05) on fortnightly body weights in ewes and similar body weights were observed in all three treatments without any significant difference. Similar to current findings, no effect (P>0.05) on live weight was observed with corn gluten meal (CGM) in beef cows [20] or dried distillers’ grains with solubles (DDGS) in ewes [21]. On contrast to the present findings, higher weight gains were reported by Abdalla *et al*. (2015) [22] in ewes with replacement of cotton seed cake (CSC) and soybean meal (SBM) by *Nigella Sativa* meal (NSM), which might be due to the presence of high unsaturated, essential fatty acids and amino acids in NSM. While, feeding of ewes with CGM as substitute to SBM decreased (P<0.05) body weights were observed in the studies of Liamadis and Milis (2007) [23], further they have reported that it could be due to low lysine and high rumen undegradable protein content in CGM compared to the SBM.

In this study, there was also no significant (P>0.05) difference in BW changes in ewes fed with block (S2) or conventional (S1) diet. Similar results were reported by Ibrahim (2008) [24] who reported no significant difference in BW changes among the ewes fed feed blocks enriched with different sources of protein and the ewes fed conventional diet. In present study, comparable BW among ewes might be related to similar composition of diets (iso-nitrogenous and iso-caloric) and DMI which was also observed by Omar *et al*. (2019) [25] and Lazarin *et al*. (2012) [26].

### Antioxidant enzyme activity

The lipid peroxidation was comparable (P>0.05) among the three dietary groups on 15^th^ day, 90^th^ day of pregnancy and 15 days before expected day of lambing (Table 3). Higher (P<0.05) lipid peroxidation values were observed during the 90^th^ day of pregnancy followed by 15 d before expected day of lambing and lower values were recorded on 15^th^ day of pregnancy. This could be explained by the fact that, 80% of foetus growth occurs in the last 2 months of pregnancy, so ewes exhibit a dramatic increase in metabolism during this period [27]. The rapid growth of foetus during the last 6 weeks of pregnancy causes an increase in fatty acid consumption from the mother’s fat reserve and production of hydrogen peroxide that has been enhanced by intense lipolisys and mobilization of fatty acids from the body deposits during pregnancy [28, 29]. Moreover, the placenta is a major source of oxidative stress because of its enrichment with polyunsaturated fatty acids [30]. Highest (P<0.05) glutathione peroxidase activity was observed on 90^th^ day of pregnancy and lowest activity at 15 days before lambing. Previous studies reported decreased concentrations of catalase, superoxide dismutase and glutathione peroxidase activities in normal pregnant ewes during late pregnancy [31, 32]. The malondialdehyde and glutathione peroxidase concentrations did not differ significantly in ewes fed S1, S2 and S3 diets on 15^th^ and 90^th^ day of pregnancy and 15 days before expected day of lambing, indicating that the inclusion of guar meal did not have any adverse effect on oxidative stress. Generally, pregnancy and lactation periods exert oxidative stress on animals even when fed traditional or untraditional diets [32]. Feeding of diet in block form also did not show any beneficial effect in decreasing the oxidative stress.

Irrespective of dietary variation, lipid peroxidation and antioxidant enzyme activities varied across the time periods of blood collection. The lipid peroxidation was higher at 90^th^ day of pregnancy and 15 days before expected day of lambing. This indicated a greater stress during these periods. Similar results were reported by Weber and Kerr (2011) [33] who observed circulating indicators of oxidative stress was not altered in pigs fed DDGS. Mohebbi-Fani *et al*. (2012) [34] also reported similar values when ewes were fed medium to low quality roughages during pregnancy. Similarly, Nawito *et al*. (2016) [35] observed that, the malondialdehyde level was increased with decreased level of superoxide dismutase in pregnant animals fed either concentrate or grazing low-quality forage.

### Serum progesterone

The progesterone concentration increased (P<0.001) with the progress of pregnancy and did not differ among the dietary treatments (P>0.05) during all the 3 blood collections (Table 3). Increase (P<0.001) in progesterone levels 15 days before expected day of lambing might be attributed to higher secretion by corpus luteum in pregnant animals. The results of the current study shown that, the partial replacement of groundnut cake with guar meal did not have any adverse effect on progesterone concentration in ewes. Further it was also observed that there was no significant (P>0.05) difference in progesterone concentration among the ewes fed conventional diet (S1) and complete feed blocks (S2 and S3). These results are in agreement with previous studies [21, 22, 36], who reported that there was no effect on progesterone profile by replacement of conventional protein supplements with unconventional protein supplements like detoxified castor seed meal or DDGS or *Nigella Sativa* meal.

### Reproductive traits

The reproductive traits of Deccani ewes are presented in Table 4. Results indicated that, the inclusion of guar meal did not show any adverse effect on estrus response, number of matings per conception, conception rate and lambing rate in ewes.

Many authors reported no effect on feeding various unconventional protein supplements on reproduction performance in animals. Erdogan *et al*. (2018) [21] observed no significant effect on inclusion of DDGS in diets on reproduction performance in ewes compared to those fed soyabean meal. In agreement with the present findings, Silva *et al*. (2015) [36] also observed no significant (P>0.05) effect on gestation length, lambing rate and litter size when ewes fed the castor seed meal or detoxified castor meal. Abdalla *et al*. (2015) [22] also observed no effect on conception rate and lambing rate by inclusion of *Nigella Sativa* meal in ewes.

In the current study, densification of complete diet did not show any significant effect on conception rate and lambing rate. Similar findings were also reported by Salman *et al*. (2017) [37] and Ibrahim (2005) [38] who observed no significant (P>0.05) improvement in conception and lambing rate in ewes fed feed blocks than conventional diet.

### Body weight of lambs

Inclusion of guar meal and physical form of feeding did not show any effect (P>0.05) on body weights of lambs at birth, on day 15 and on day 30 (Table 5). Similarly, Silva *et al*. (2014) [39] observed no significant effect on the weight of the lambs from birth to weaning with supplementation of detoxified castor meal in the diet of ewes during pregnancy. Erdogan *et al*. (2018) [21] also reported that, ewe nutrition had no significant effect on lamb weight at birth, when the ewes supplemented with soyabean meal and DDGS. On the other hand, Kennedy *et al*. (2015) [40] found the addition of DDGS to cattle rations during the last period of gestation provided significant advantages in terms of calf birth weight and weaning weight attributed to the high protein content of DDGS. While, Abdalla *et al*. (2015) [22] recorded significantly (P<0.05) higher birth weight of lambs born to the ewes fed *Nigella Sativa* meal compared to control group. The higher birth weight of lambs born to ewes fed on *Nigella Sativa* meal might be related to that protein requirements were adequate and rapidly degradable.

In the present study, physical form of the diet did not show any significant (P>0.05) effect on lamb birth weights. Similar findings were reported by Ibrahim (2008) [24] who observed no effects on birth weight of lambs with supplementation of feed blocks enriched with cotton seed meal.

## Conclusion

The conventionally used groundnut cake could be satisfactorily replaced by guar meal up to 50% on nitrogen basis in sorghum stover based complete feed blocks without any adverse effect on antioxidant status, progesterone concentration, conception rate, non return rate, no of matings per service, lambing rate, body weights of lambs at birth, 15 and 30 days of age. Further it was observed that densification of complete diets had no effect (P>0.05) on the conception rate and lambing rate in ewes.

## Supporting information

S1 Data (XLSX)
